# Pharmacokinetics and the optimal regimen for levofloxacin in critically ill patients receiving continuous hemodiafiltration

**DOI:** 10.1186/s40560-015-0089-0

**Published:** 2015-05-08

**Authors:** Takeshi Wada, Masaki Kobayashi, Yuichi Ono, Asumi Mizugaki, Kenichi Katabami, Kunihiko Maekawa, Daisuke Miyamoto, Yuichiro Yanagida, Mineji Hayakawa, Atsushi Sawamura, Ken Iseki, Satoshi Gando

**Affiliations:** Division of Acute and Critical Care Medicine, Department of Anesthesiology and Critical Care Medicine, Hokkaido University Graduate School of Medicine, N15W7, Kita-ku, Sapporo, 060-8638 Japan; Laboratory of Clinical Pharmaceutics & Therapeutics, Division of Pharmasciences, Faculty of Pharmaceutical Sciences, Hokkaido University, N12W6, Kita-ku, Sapporo, 060-0812 Japan; Department of Pharmacy, Hokkaido University Hospital, N14W5, Kita-ku, Sapporo, 060-8648 Japan

**Keywords:** Levofloxacin, Pharmacokinetics, Continuous hemodiafiltration, Clearance

## Abstract

The aim of this study was to establish the pharmacokinetics of levofloxacin (LVFX) and determine the optimal dose of this drug in critically ill patients receiving continuous hemodiafiltration (CHDF). The results of *in vivo* and *in vitro* studies showed the pharmacokinetics of LVFX total clearance (CL_total_) according to the creatinine clearance (CL_Cre_), dialysate flow (Q_D_), and ultrafiltrate flow (Q_F_), to be as follows: CL_total_ (l/h) = 0.0836 × CL_Cre_ (ml/min) + 0.013 × body weight (kg) + 0.94(Q_D_ + Q_F_) (l/h). The optimal dose of LVFX was expressed by the following formula: 50 × CL_total_. These results demonstrate that the usual dose of LVFX (500 mg) was sufficient for the patients evaluated in this study.

## Findings

The pharmacokinetics of levofloxacin (LVFX) total clearance (CL_total_) were determined based on the creatinine clearance (CL_Cre_), dialysate flow (Q_D_), and ultrafiltrate flow (Q_F_), as follows:

$$ {\mathrm{CL}}_{\mathrm{total}}\left(\mathrm{l}/\mathrm{h}\right)=0.0836\times {\mathrm{CL}}_{\mathrm{Cre}}\left(\mathrm{ml}/ \min \right)+0.013\times \mathrm{body}\;\mathrm{weight}\left(\mathrm{kg}\right)+0.94\left({\mathrm{Q}}_{\mathrm{D}}+{\mathrm{Q}}_{\mathrm{F}}\right)\left(\mathrm{l}/\mathrm{h}\right) $$

## Introduction

Critically ill patients often require continuous hemodiafiltration (CHDF) as a result of acute kidney injury induced by severe sepsis. Levofloxacin (LVFX) is widely used for treatment in these patients. However, the pharmacokinetics (PK) of LVFX during CHDF are not uniform, as CHDF is performed using various combinations of the dialysate flow (Q_D_) and ultrafiltrate flow (Q_F_). The aim of the present study was to estimate the PK of LVFX in patients receiving CHDF and determine the optimal dose of LVFX for this patient population.

## Methods

Approval for this study was obtained from the institutional review board, − The Ethics Committee of Hokkaido University School of Medicine (011–0107). Informed consent for this study was obtained from the patients’ next of kin.

### *In vitro study*

A CHDF circuit model (JUN-600, JUN-KEN MEDICAL Co., Tokyo, Japan) was established using a cellulose triacetate hollow fiber 1.1 m^2^ hemofilter (UT-1100, Nipro, Japan). The machine was primed with fresh frozen plasma (FFP), and 100 mg of LVFX were added to the circuit. The FFP flow was fixed at 150 ml/min, and the CHDF conditions were as follows: the Q_D_ was defined from 0, 1, and 2 l/h; the Q_F_ was defined from 0, 1, and 2 l/h, independent of Q_D_. Samples were obtained from the prehemofilter and *ultrafiltrates* at 15, 30, 45, and 60 min after the start of CHDF. The sieving coefficient (SC) values were calculated based on the LVFX concentrations in the filtrates and prehemofilter. The levels of clearance (CL) *via* CHDF (CL_CHDF_) were obtained for the product of SC and (Q_D_ + Q_F_) and then were plotted, respectively.

### *In vivo study*

Four patients with acute kidney injury were administered LVFX during CHDF (ACH-Σ, Asahi Kasei Medical. Co., Tokyo, Japan). The hemofilter used in the *in vivo* study was a polysulfone hollow fiber 1.3 m^2^ hemofilter (EXCELFLO AEF-13, Asahi Kasei Medical. Co., Tokyo, Japan). Replacement fluid was connected to the post-filter blood line. The 24 h creatinine clearance values were accurately measured based on the urine and serum creatinine levels and the 24 h urine output. The LVFX dose was set at 500 mg/day for all patients. Blood samples were collected before the administration of LVFX and at 1, 2, 6, 12, and 24 hours after the start of drug administration. The concentration of LVFX was determined according to a high-performance liquid chromatography method, and a pharmacokinetic analysis was performed using a nonlinear least-squares regression program. The parameters were calculated by employing a two-compartment open model with a constant rate of infusion. The area under the concentration-time curve (AUC) was determined based on the trapezoidal rule. The optimal dose of LVFX was calculated based on the following relational expression:

$$ {\mathrm{CL}}_{\mathrm{total}}=\mathrm{dose}\;\mathrm{of}\;\mathrm{drug}/\mathrm{A}\mathrm{U}\mathrm{C} $$

## Results

**Figure 1:**
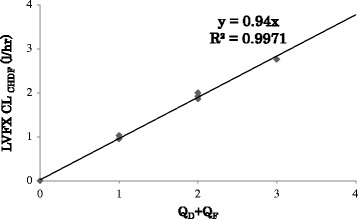
Pharmacokinetics of levofloxacin (LVFX) clearance (CL) during continuous hemodiafiltration. A simple linear regression analysis revealed a strong correlation between LVFX CL_CHDF_ and Q_D_ + Q_F_.

The CL_CHDF_ obtained *via* interpolation into a simple linear regression of CL_CHDF_ against (Q_D_ + Q_F_) closely correlated with the experimental data (Figure 1). The PK of LVFX clearance (CL_vivo_) was determined based on the creatinine clearance (CL_Cre_) and body weight (BW), according to previous study [1]. The LVFX total clearance (CL_total_) in a patient receiving CHDF was calculated as follows:

$$ {\mathrm{CL}}_{\mathrm{total}}\left(\mathrm{l}/\mathrm{h}\right)={\mathrm{CL}}_{\mathrm{vivo}}+{\mathrm{CL}}_{\mathrm{CHDF}} $$

**Figure 2:**
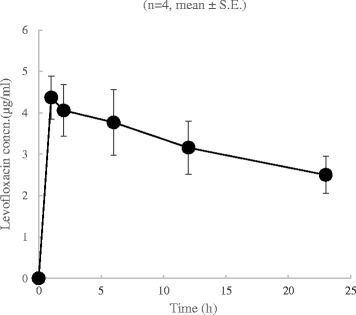
Pharmacokinetics of the levofloxacin (LVFX) concentration-time curve during LVFX administration of 500 mg first 24 hours. Cons, concentration. SE, standard error.

The values of predictive CL_total_ were calculated based on this formula. Table 1 shows the characteristics of the patients. We were unable to calculate the predictive CL_total_ in patient No. 3 because the urine creatinine level was not examined in this case. The LVFX concentration-time curve is shown in Figure 2, and the pharmacokinetic parameters of LVFX are presented in Table 2. The AUC was 73.9 ± 13.8 (mg/l h).

**Table 1:** Characteristics of the patients

**Patient**	**Sex**	**Age (years)**	**Diagnosis**	**Weight (kg)**	**APACHE II**	**The cause of AKI**	**The value of Cre on admission to ICU (mg/dl)**	**Duration of CHDF (days)**
1	Male	75	Ruptured AAA	70.7	31	Hemorrhagic shock	1.32	66
2	Male	59	OHCA	88.9	42	PCAS	0.98	5
3	Male	46	Congenital heart disease	50.0	21	Major cardiac operation	0.69	45
4	Male	58	ML	58.3	41	Drug induced	2.04	38
Mean ± SE		59.5 ± 6.0	-	70.0 ± 8.5	33.8 ± 4.9	-	1.25 + 0.29	38.5 + 12.7
**BUN (mmol/l)**	**24 h CL**_**cre**_ **(ml/min)**	**Q**_**D**_ **(l/h)**	**Q**_**F**_ **(l/h)**	**Predictive CL**_**total**_ **(l/h)**	**Calculated optimal dose of LVFX (mg)**			
42	3.4	1.0	2.0	4.0	200			
43	0.9	1.0	2.0	4.0	200			
40	-	1.0	2.0	-	-			
32	3.4	0.5	1.5	2.9	145			
39.3 ± 5.0	2.6 ± 0.8	0.86 + 0.13	1.88 + 0.13	3.6 ± 0.4	182 + 18.3			

AAA abdominal aortic aneurysm, OHCA out-of-hospital cardiac arrest, ML malignant lymphoma, APACHE II Acute Physiology and Chronic Health Evaluation II score, AKI acute kidney injury, PCAS post-cardiac arrest syndrome, Cre creatinine, ICU intensive care unit, CHDF continuous hemodiafiltration, BUN blood urea nitrogen, CL_cre_ creatinine clearance, CL_total_ total clearance, QD dialysate flow, QF ultrafiltrate flow, LVFX levofloxacin, SE standard error.

**Table 2:** **Pharmacokinetic parameters of levofloxacin in the patients receiving continuous hemodiafiltration**

**Patient**	**CL**_**total**_ **(l/h)**	**t**_**1/2**_ **(h)**	**C**_**max**_ **(mcg/ml)**	**AUC [(mg/l) h]**
1	4.62	13.1	5.7	108.3
2	12.3	14.4	3.0	40.8
3	6.64	28.9	4.7	75.3
4	7.01	11.4	4.4	71.3
Mean ± SE	7.63 + 1.6	16.9 + 4.0	4.5 + 0.6	73.9 + 13.8

*CL*_*total*_ total clearance, *t1/2* a half-life, *C*_*max*_ maximum concentration, *AUC* area under the concentration-time curve, *SE* standard error.

## Discussion

The ratio of AUC/minimum inhibitory concentration (MIC) is a well-known important PK and pharmacodynamics predictor of the clinical efficacy of fluoroquinolones, including LVFX. Previous studies suggest that the AUC/MIC of ≥100 (h) is required in compromised patients or those exhibiting severe Gram-negative rod or staphylococcal infection [2-4]. In addition, the MIC for 90% of tested strains against most common Gram-negative aerobic pathogens is < 0.5 (μg/ml) [5]. Therefore, we determined the target AUC to be ≥ 50 and the optimal dose of LVFX to be 50 × CL_total_. Hence, the LVFX concentrations reached higher than optimal concentrations, and infection could therefore be successfully controlled in these patients.

Three factors affect the PK during CHDF as follows: 1) pore size and protein binding fraction of the drug; 2) molecular size; 3) Q_D_ and Q_F_ in the CHDF protocol [6]. The triacetate and polysulfone membranes used in this study have large pores and do not have a capacity for drug absorption, characteristics recommended for CHDF. The molecular size of LVFX is 361 Da, which is less than that of ciprofloxacin (CPFX) (368 Da). The results of our previous study suggested that the pore size of the hemofilter does not influence the CL_CHDF_, likely due to the sufficiently low molecular weight of CPFX [7]. *This previous* study also indicated that the surface area of the hemofilter with a large amount of Q_D_ possibly affects the clearance of small solutes, such as fluoroquinolones [7]. Therefore, the current results are not applicable in cases in which the Q_D_ is large.

The limitations of this study should be addressed. First, the results of a study by Takigawara et al. [1], showing the relationship between the PK of LVFX and the CL_Cre_, were based on patients with a normal renal function. These results are not applicable to the present study, as we included patients with more severe kidney injury. Second, the current study included a very small number of patients. Therefore, a larger, more precise clinical study is needed to confirm our results.

## Abbreviations

LVFX: levofloxacin
CHDF: continuous hemodiafiltration
CL_Cre_: creatinine clearance
Q_D_: dialysate flow
Q_F_: ultrafiltrate flow
CL_total_: LVFX total clearance
PK: Pharmacokinetics
FFP: fresh frozen plasma
SC: sieving coefficient
CL_CHDF_: clearance by CHDF
CL_*vivo*_: clearance in patients
BW: body weight
AUC: area under the concentration-time curve
MIC: minimum inhibitory concentration
CPFX: ciprofloxacin
